# Alkaloids in Marine Algae

**DOI:** 10.3390/md8020269

**Published:** 2010-02-04

**Authors:** Kasım Cemal Güven, Aline Percot, Ekrem Sezik

**Affiliations:** 1 Istanbul Aydın University, Inönü Cad., 40, Sefaköy, Istanbul, Turkey; 2 Laboratoire de Dynamique, Interactions et Réactivité (LADIR), UMR 7075 CNRS–UPMC Univ Paris 06, 2 rue Henry Dunant, 94320 Thiais, France; E-Mail: aline.percot@gmail.com; 3 Department of Pharmacognosy, Hipodrom Ankara, Faculty of Pharmacy, Gazi University, Turkey; E-Mail: ekrmsezik@gmail.com

**Keywords:** alkaloids, phenylethylamine alkaloids, indole alkaloids, halogenated indole alkaloids, other alkaloids

## Abstract

This paper presents the alkaloids found in green, brown and red marine algae. Algal chemistry has interested many researchers in order to develop new drugs, as algae include compounds with functional groups which are characteristic from this particular source. Among these compounds, alkaloids present special interest because of their pharmacological activities. Alkaloid chemistry has been widely studied in terrestrial plants, but the number of studies in algae is insignificant. In this review, a detailed account of macro algae alkaloids with their structure and pharmacological activities is presented. The alkaloids found in marine algae may be divided into three groups: 1. Phenylethylamine alkaloids, 2. Indole and halogenated indole alkaloids, 3. Other alkaloids.

## 1. Introduction

The term alkaloid was first proposed by Meissner in 1819 to characterize these “alkali-like” compounds found in plants [1,2], but it was not precisely defined [3]. With time, the definition has changed [4] to: a compound that has nitrogen atom(s) in a cyclic ring. Numerous biological amines and halogenated cyclic nitrogen-containing substances are included in the term alkaloid. The latter is specific from marine organisms and marine algae. They could not be found in terrestrial plants.

Some alkaloids isolated from marine algae correspond to contaminants, such as the indole derivative communesin isolated from a *Penicillium* **s**p. found on the green alga *Enteromorpha intestinalis* [5] and leptosins from *Leptosphaeria* on *Sargassum tortillae* [6]. These alkaloids were improperly attributed to algae and were not included in this paper.

After the isolation of alkaloids, pure active compounds were used in therapy instead of plant extracts. Isolation of active compounds from plants began in 18th century. Morphine was the first alkaloid extracted from a terrestrial plant in 1805 as reported by Kappelmayer [7] and hordenine was the first alkaloid isolated from a marine algae in 1969 [8,9]. Today approximately two thousand alkaloids are known. They occur abundantly in terrestrial plants and rarely in marine algae.

In this chapter alkaloids in marine algae were classified in three groups as follows:

1. Phenylethylamine alkaloids.
2. Indole and halogenated indole alkaloids.
3. Other alkaloids.

## 2. Phenylethylamine Group

### 2.1. Phenylethylamine (PEA)

**Figure 1:**
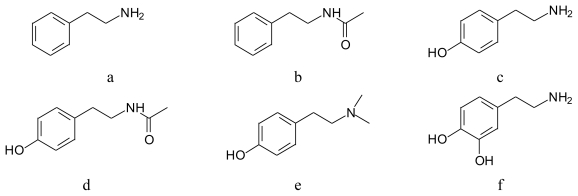
Structures of phenylethylamine derivatives: (a) PEA; (b) N-ACPEA; (c) TYR; (d) N-ACTYR; (e) HORD; (f) DOP.

PEA (β/2-phenylethylamine, phenethylamine) is an aromatic amine made up of a benzene ring to which an ethylamine side chain is attached (Figure 1a). The PEA alkaloid group includes important alkaloids. It is a precursor of many natural and synthetic compounds. Several substituted PEAs are pharmacologically active compounds found in plants and animals. This group includes simple phenylamine (tyramine, hordenine) and catecholamine (dopamine). The latter was found in animals and terrestrial plants [10]. The structure of PEA allows substitutions on the aromatic ring, the α and β carbons and terminal amino group. The published papers concern amine compounds in marine algae [11,12], and in the plant kingdom including algae [13].

*Sources*: Some brown marine algae containing PEA are [11]: *Desmerestia aculeata*, *Desmerestia viridis;* Red: *Ceramium rubrum*, *Cystoclonium purpureum*, *Delesseria sanguine*, *Dumontia incrassata*, *Polysiphonia urceolata*, *Polyides rotundus.* Recently the presence of PEA was examined in 17 marine algae and it was found only in six red algae [14]: *Gelidium crinale*, *Gracilaria bursa-pastoris*, *Halymenia floresii*, *Phyllophora crispa*, *Polysiphonia morrowii*, *Polysiphonia tripinnata.* PEA was also found in the microalgae *Scenedesmus acutus* [15].

*Pharmacological activity*: PEA in the human brain acts as a neuromodulator and a neurotransmitter. PEA has been shown to relieve depression in 60% of depressed patients. It has been proposed that a PEA deficit may be the cause of a common form of depressive illness [16]. Substituted PEAs are pharmacologically active compounds as hormones, stimulants, hallucinogens, entactogenes, anorectics, bronchodilators and antidepressants [17].

*N*-acetylphenylethylamine (*N*-ACPEA, *N*-(2-phenylethylacetamide; Figure 1b)

*Source: N*-acetylphenylethylamine was first isolated from the red algae *Phyllophora crispa* and *Gelidium crinale* [18].

*Pharmacological activity: N*-ACPEA induced also rotations ipsilateral to the side of the brain lesion as PEA but its activity was 90% less active than β-PEA [16].

### 2.2. Tyramine (TYR, 4-hydroxyphenylethylamine; Figure 1c)

TYR is a monoamine derivative of the amino acid tyrosine.

*Source*: TYR occurs widely in plants, fungi and animal but is rare in algae. It was detected in the brown alga *Laminaria saccharina*, and red algae *Chondrus crispus* and *Polysiphonia urceolata* [19] and in the microalgae *Scenedesmus acutus* [14].

*Pharmacological activity*: TYR is a pharmacologically important compound. It stimulates the central nervous system, causes vasoconstriction, increases heart rate and blood pressure and is also responsible for migraines.

*N*-Acetyltyramine (*N*-ACTYR; Figure 1d)

Acetyl derivative of tyramine

*Source*: It was found in the marine algae *Phyllophora crispa* and *Gelidium crinale* [18] and is produced by many microorganisms [14] and terrestrial plants [20].

*Pharmacological activity*: N-ACTYR is a neuropeptide and an important amine for chemical and pharmacological purposes. The presence of urinary N-ACTYR in neuroblastoma patients was demonstrated [21].

### 2.3. Hordenine (Anhaline) (HORD, 4-(2-dimethylaminoethyl) phenol; Figure 1e)

It was first isolated from terrestrial plant *Anhanolium fissuratus* in 1894 [22] and its structure was elucidated in 1906 [23].

*Source*: HORD was first obtained from red algae *Phyllophora nervosa* [new name: *Phyllophora crispa*] [8a,b], and later from *Ahnfeltia paradoxa* [24], from *Gigartina stellata (Mastocarpus stellatus)* [25] and from *Gelidium crinale* [26]. The amount of HORD was determined in *Gelidium crinale* [26] and *Phyllophora nervosa* [27] as 9.54–39.66 μg/g, respectively.

*Pharmacological activity*: The roles of amine compounds in marine algae are not clear [28]. HORD is diuretic and affects the central nervous system. In the past, HORD was used for the treatment of diarrhea and dysentery [29]. It has a positive inotropic effect upon the heart, increases systolic and diastolic blood pressure, peripheral blood volume and inhibits gut movement [30]. All effects are short and only observable with high doses.

### 2.4. Dopamine (DOP, 3,4-dihydroxyphenethylamine; Figure 1f)

DOP is a catecholamine carrying two hydroxyl groups in the position 3 and 4 of the phenyl ring. It is produced in the organism by decarboxylation of dihydroxyphenylalanine.

*Source*: DOP was found in animals and several terrestrial plants [9] and only one reference mentions its presence in the green alga *Monostroma fuscum* [31].

*Pharmacological activity*: It is a hormone and a neurotransmitter. DOP is a sympathomimetic compound. It was used to treat cardiovascular and kidney disorders [32].

## 3. Indole Group

This alkaloid group containing a benzylpyrrole (derived from tryptophan) includes caulerpin, caulersin, fragilamide, martensine, martefragine, denticine and almazolone.

### 3.1. Caulerpin (CLP, dimethyl-6,13-dihydrodibenzo [b,i]phenazine-5,12-dicarboxylate methyl ester; Figure 2)

Caulerpin contains two indole groups linked by a cyclic ring containing eight carbons with two carboxy groups. The structure of CLP (I) was first proposed [33,34] and later revised [35]. Its crystal structure [36] was studied. Two CLP (I) analogues CLP (II) and (III) were also isolated from *Caulerpa racemosa* [37].

*Source*: CLP (I) was isolated especially from green algae and some from red algae. CLP (I) was first extracted from *Caulerpa racemosa*, *C. sertularioides*, *C. serrulata* [33,34] and later, isolated from various *Caulerpa* sp. as: *C. lamourouxii* [38], *C. racemosa var. macrophysa*, *C. racemosa var. laetevirens*, *C. ashmeadii* [39], *C. cupressoides*, *C. paspaloides*, *C. prolifera*, *C. sertularioides* [40], *C. peltata* [40–42], *C. racemosa var. clavifera* [43], *C. taxifolia* [44,45], *C. serrulata* [33]. CLP (I) was also isolated from other algae: green; *Codium decorticatum* [46], *Halimeda incrassate* [47], and red; *Laurencia majuscula* (CLP I, II) [48], *Hypnea concornis* [48], *Caloglossa leprieurii* [48,49], *Chondria armata* [50].

The content of CLP (I) in *Caulerpa* sp. are 15% for *C. lentilifera*, 5% for *C. rasemosa*, 2% for *C.microphysa* and 8% for *C. sertulorides* [51]. *C. taxifolia* has bloomed explosively in the Mediterranean Sea and has become a major ecological problem [52].

*Pharmacological activity*: There are different opinions on the toxicity of CLP (I). Symptoms were observed after the ingestion of *Caulerpa* genus [33]. It shows low toxicity [43]. *C. racemosa* extracts showed some cytotoxicity, but CLP (I) isolated from these extracts did not show any activity [53]. CLP (I) exhibited a moderate *in-vitro* antitumor activity against crown gall tumor [54]. CLP (I) showed moderate antibacterial activity against 8 species of bacteria isolated from algal surface [51]. CLP (I) containing alga *Laurencia majuscula* showed antifungal activity [55]. CLP (I) has been shown to be a plant growth regulator [55–57]. CLP (I) showed no peroxidase activity [58].

### 3.2. Caulersin (CLS)

**Figure 3:**
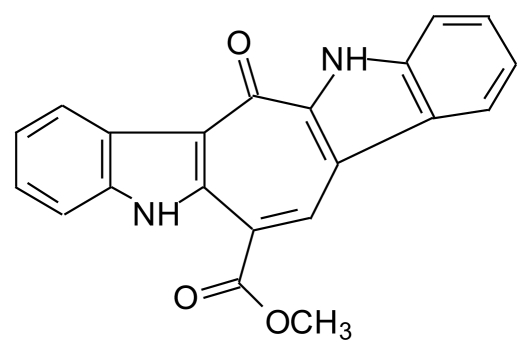
Structure of CLS.

CLS is a bisindole alkaloid with a 7 members central ring and two ≪anti parallel≫ indole cores [59] (Figure 3). It was synthesized by several authors [60–63]. CLS has three isomers: A, B and C [62].

*Source*: CLS was isolated from *Caulerpa serrulata* [59].

### 3.3. Martensia fragilis alkaloids

Several compounds were isolated from *Martensia fragilis (M. denticulata)* such as: fragilamide, martensines, martefragin A, and denticins.

#### 3.3.1. Fragilamide (FRG)

**Figure 4:**
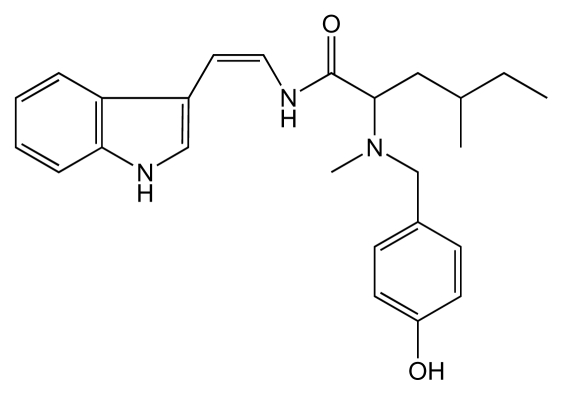
Structure of FRG.

FRG was extracted from the red alga *Martensia fragilis.* It is a labile amine and it rapidly auto-oxidized in solution. FRG is a 3-substituted indole and corresponds to a *N*-methylhomoisoleucyl unit and a *p*-hydroxybenzyl group connected to the indole unit C-3. The amide NH was connected to a cis disubstituted carbon-carbon double bond [64] (Figure 4).

*Pharmacological activity*: FRG showed strong antioxidant activity [65].

#### 3.3.2. Martensines (MRT)

MRT A and B were extracted from the red algae *Martensia fragilis* [64]. MRTs are 3-substituted indoles.

##### 3.3.2.1. Martensine A

**Figure 5:**
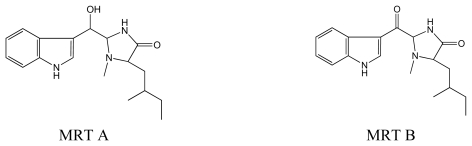
Structures of MRT A and MRT B.

MRT A is a 3- substituted indole bound to a 5-membered lactam ring [64] (Figure 5).

*Pharmacological activity*: MRT A shows an antibiotic activity against *Bacillus subtilis*, *Staphylococcus aureus*, and *Mycobacterium smegmatis* [64].

##### 3.3.2.2. Martensine B

MRT B contains two carbonyl as γ-lactam and an aryl ketone group [64] (Figure 5).

#### 3.3.3. Martefragin A (MRF A)

**Figure 6:**
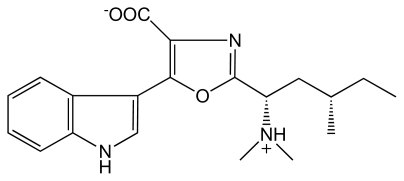
Structures of MRF A.

MRF A was isolated from *Martensia fragilis*. MRF A displays a 3-oxazolylindole structure [66 a,b] (Figure 6). It was also synthesized [67].

*Pharmacological activity*: MRF A showed inhibitory activity on NADPH- depending lipid peroxidation in rat liver microsomes [66,67].

#### 3.3.4. Denticins (DTC)

**Figure 7:**
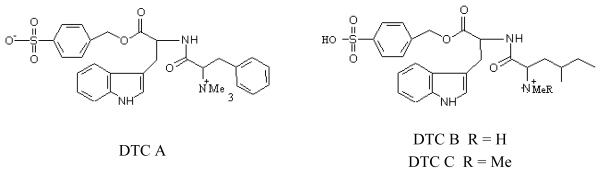
Structures of DTCs.

DTC’s were isolated from *Martensia denticulata* [68]. DTC(s) are 3-subsituted indole derivates named DTC A, B and C. These alkaloids contain sulfonic acids which are rarely found in alkaloids (Figure 7).

*Pharmacological activity*: DTC(s) have an anti-photo-oxidative activity [68].

### 3.4. Almazolone (ALM)

**Figure 8:**
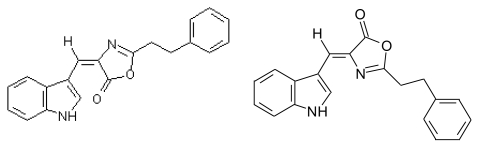
Structures of ALM isomers E and Z.

ALM was isolated from the red alga *Haraldiophyllum* sp. collected in Dakar (Senegal). ALM is a disubstituted oxazolindole derivate. It has two steroisomers, which correspond to synthesized *E* and *Z* isomers [69] (Figure 8).

## 4. Halogenated Indole Alkaloids (HLI)

HLI alkaloids were isolated only in marine organisms and algae but not in terrestrial plants. Many HLI alkaloids were isolated from red algae and only one from a green alga. These alkaloids contain an indole group substituted by bromine and chlorine atoms. Sulfur-containing bromoalkaloids were also extracted from red algae.

*Pharmacological activity*: Antibacterial activities of halogenated alkaloids were examined on terrestrial and some marine bacteria.

### 4.1. Bromoindole

#### 4.1.1. Bromoindoles and *N*-methylbromoindoles isolated from algae are given by the source

**Figure 9:**
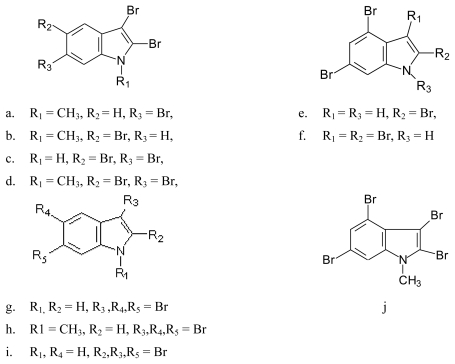
Structures of bromo compounds isolated from red algae *Laurencia brongniartii*, *Laurencia similis* and *Laurencia decumbens.*

1. Red alga *Laurencia brongniartii* collected from Caribbean Sea [70] and Okinawan Sea [71] (Figure 9): 2,3,6-tribromo-1-methyl indole (**9a**) [70], 2,3,5-tribromo-1-methyl indole (**9b**) [72], 2,3,5,6-tetrabromo-1*H*-indole (**9c**) [70], 2,3,5,6-tetrabromo-1-methyl indole (**9d**) [70], 2,4,6-tribromo-1*H*-indole (**9e**)[71] and 2,3,4,6-tetrabromo-1*H*-indole (**9f**) [71]. Compounds **9c** and **9f** were also identified in the red alga *Laurencia similis* collected from Pulau Gaya, Malaysia [72] and compound **9b** was isolated from a red alga *Laurencia decumbens* collected from Weizhou Island (South China-Sea) [73].
  *Pharmacological activity*: Among compounds **9a**–**d** only **9c** showed antibacterial activity against *Bacillus subtilis* and *Saccharomyces cerevisiae* [70].
2. Red alga *Laurencia similis* collected from Sanya, China [74] (Figure 9): 3,5,6-tribromo-1*H*-indole (**9g**) [74], 3,5,6-tribromo-1-methylindole (**9h**) [74] and 2,3,6-tribromo-1*H*-indole (**9i**) [74]
3. Bromoindoles isolated from the red alga *Laurencia decumbens* collected from Weizhou Island (South China- Sea) [73] (Figure 9): 2,3,4,6-tetrabromo-1-methylindole (**9j**).

### 4.2. Sulfur-containing bromoalkaloids isolated from Laurencia brongniartii

**Figure 10:**
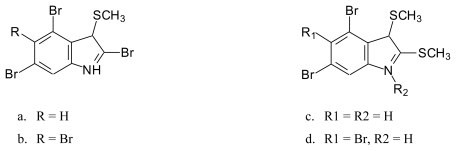
Structures of thiobromo compounds isolated from the red alga *Laurencia brongniartii.*

**Figure 11:**
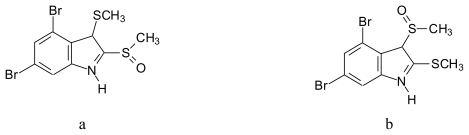
Structures of thiomethyl and sulfoxide containing bromoindoles isolated from *Laurencia brongniartii*.

- Thiobromoindoles [73] (Figure 10): 3-thiomethyl 2,4,6-tribromo-1*H*-indole (**10a**), 3-thiomethyl 2,4,5,6- tetrabromo-1*H*-indole (**10b**), 2,3-dithiomethyl-4,6-dibromo-1*H*-indole (**10c**) and 2,3-dithio-methyl-4,5,6- tribromo-1*H*-indole (**10d**).
- Thiomethyl and sulfoxide containing bromoindoles [73] (Figure 11): 2-thiomethyl-3-sulfoxymethyl-4,6-dibromoindole (**11a**) and 2-sulfoxymethyl-3-thiomethyl-4,6-dibromo-1*H*-indole (**11b**).

*Laurencia brongniartii* is an exceptionally important red algae. The isolated compounds were:

1. Four sulfur-containing bromoindoles collected from Caribbean Sea [70,71].
2. Four other sulfur-containing bromoindoles isolated from the Taiwanese coast [75], Okinawan Sea [71].
3. Six new indoles of which the two are sulfoxides from the Okinawan Sea [71].
4. A bisindole collected in Okinawan waters [71].

These results showed that halogenated alkaloid types depended on the same algae and collection areas [71].

### 4.3. Polyhalogenated indoles

**Figure 12:**
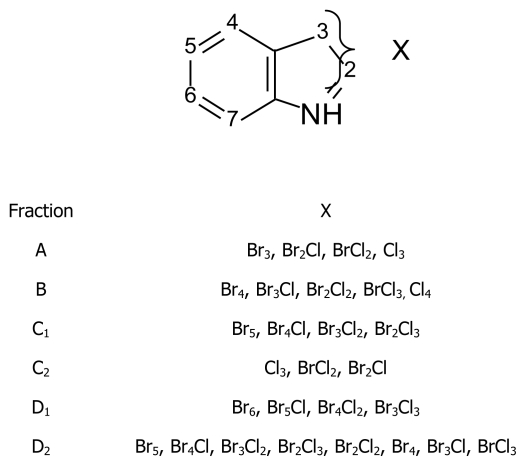
Structure of polyhalogenated indoles from *Rhodophyllis membranacea*.

Many polyhalogenated indoles were identified in *Rhodophyllis membranacea* collected from the Kaikoura coast (New Zealand). The fractions obtained from the extract of *R. membranacea* contain polychlorinated and polybrominated alkaloids [75] (Figure 12).

*Pharmacological activity*: Crude extract of *R. membranacea* showed strong antifungal activity due to the presences of these polyhalogenated indoles [75].

### 4.4. Bromobisindole

#### 4.4.1. Polyhalogenated bisindoles (Figure 13)

4,4′-Dichloro-5,5′-dibromo-7,7′-dimethoxy-3,3′-bis-1*H*-indole (**13a**) was identified from the green alga *Chaetomorpha basiretorsa* [76]. 2,2′,5,5′,6,6′-hexabromo-3,3′-bis-1*H*-indole (**13b**) was identified from *Laurencia similis* collected from the coast of Sanya, Hainan Island (China) [77].

#### 4.4.2. Thiomethyl-containing bromobisindoles

**Figure 14:**
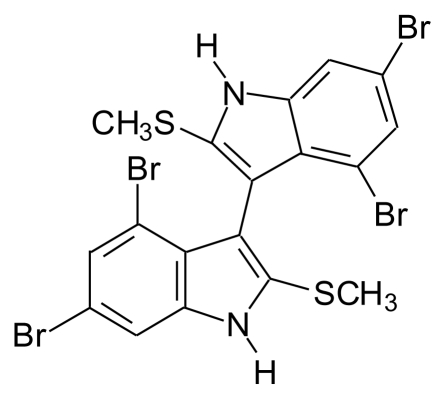
Structure of thiomethyl containing bromobisindole.

3,3′-bis(4,6-Dibromo-3-methylthio) indole was isolated from *Laurencia brongniartii* collected in Okinawan Sea [71] (Figure 14).

## 5. Other

### 5.1. Lophocladines (LO, Figure 15)

**Figure 15:**
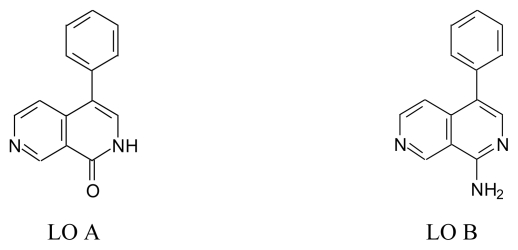
Structure of LO A and LO B.

There are two derivatives: lophocladine A and lophocladine B which were isolated from a red alga *Lophocladia* sp. collected from Fijian Island (New Zealand) [78] (Figure 15).

1. Lophocladines A (LO A, 4-phenyl-[2,7]-naphthyridine-1(2*H*)-one, **15a**)
2. Lophocladines B (LO B, 4-phenyl-[2,7]-naphtyridine-1-amine, **15b**)

*Pharmacological activity*: The cytotoxic activities of LO A and B were investigated on NCI-H-460 lung cancer and neuro-2a neuroblastoma and MDA-MB-435 breast cancer lines. Only LO B showed moderate cytotoxic activity on MDA-MB-435 and NCI-H-460 cell lines but not on neuro 2-a cell [78].

## 6. Conclusions

Marine algal alkaloids have been reviewed in this paper. Structurally the alkaloids isolated from marine algae mostly belong to the phenylethylamine and indole groups. Biological activities of these alkaloids were not wholly investigated. Alkaloids of marine algae are relatively rare, when compared with terrestrial plant alkaloids. Research on marine drugs has largely focused on finding drugs for cancer treatment. Nowadays, no alkaloids obtained from marine algae are used in medicine.

## Figures and Tables

**Figure 2:**
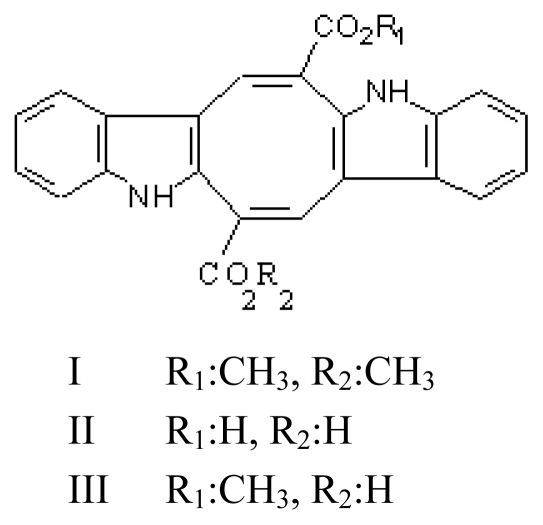
Structures of CLP analogues (I, II, III).

**Figure 13:**
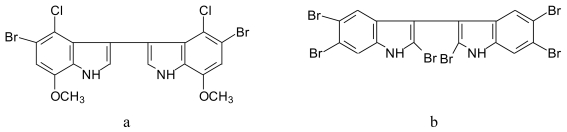
Structure of bromobisindoles isolated from *Chaetomorpha basiretorsa* and *Laurencia similis*.
